# A versatile LC-MS/MS approach for comprehensive, quantitative analysis of central metabolic pathways

**DOI:** 10.12688/wellcomeopenres.14832.1

**Published:** 2018-09-20

**Authors:** Adhish Walvekar, Zeenat Rashida, Hemanth Maddali, Sunil Laxman

**Affiliations:** 1 Institute for Stem Cell biology and Regenerative Medicine (inStem), Bangalore, Karnataka, 560065, India; 2 Manipal Academy of Higher Education, Manipal, Karnataka, 576104, India

**Keywords:** Mass spectrometry, central carbon metabolism, OBHA derivatization, stable-isotope labeling, labeling of amino acids and nucleotides, kinetics of label incorporation, metabolic flux analysis

## Abstract

Liquid chromatography-mass spectrometry (LC-MS/MS) based approaches are widely used for the identification and quantitation of specific metabolites, and are a preferred approach towards analyzing cellular metabolism. Most methods developed come with specific requirements such as unique columns, ion-pairing reagents and pH conditions, and typically allow measurements in a specific pathway alone. Here, we present a single column-based set of methods for simultaneous coverage of multiple pathways, primarily focusing on central carbon, amino acid, and nucleotide metabolism. We further demonstrate the use of this method for quantitative, stable isotope-based metabolic flux experiments, expanding its use beyond steady-state level measurements of metabolites. The expected kinetics of label accumulation pertinent to the pathway under study are presented with some examples. The methods discussed here are broadly applicable, minimize the need for multiple chromatographic resolution methods, and highlight how simple labeling experiments can be valuable in facilitating a comprehensive understanding of the metabolic state of cells.

## Introduction

A quantitative and sensitive detection of steady-state metabolites, as well as metabolic flux analysis (MFA) in cells is a pressing analytical requirement in modern biology. Different metabolites are decorated with different functional groups. Exploiting the unique features of distinct metabolites, several methods using liquid chromatography-based mass spectrometry (LC-MS/MS) have been developed and used widely for their reliable detection. However, typically multiple different methods (either involving different chromatographic separation methods including ion-pair coupling methods ^1^, or different derivatization protocols ^2– 4^) are used to detect and quantify different pools of metabolites. Each set of metabolites have challenges in either separation by chromatography, or ionization and detection by mass spectrometry, or both. For example, the tricarboxylic acid (TCA) cycle intermediates can be detected either without derivatization on a HILIC column ^5^, or by derivatization of these molecules to permit resolution and detection ^2, 6^. Similarly, amino acids can be detected either without derivatization ^7^ or by incorporating ion-pairing reagents ^1, 8, 9^ or by derivatization ^4^. As a result, currently most methods used typically execute metabolite measurements in individual pathways, focusing on single pathways such as glycolysis, the pentose phosphate pathway (PPP) or the TCA cycle. Further, MFA experiments (typically with labeled glucose) detect label incorporation only in these pathways. Comprehensive, reliable methods that can simultaneously cover multiple metabolic pathways and their important branch points are currently lacking. Their development can substantially simplify the execution of steady-state and MFA analysis of central metabolism. Here we present a versatile set of LC-MS/MS methods using a single column for metabolite separation, and use of a single derivatization agent, completely avoiding ion-pair coupling based separation, for a comprehensive analysis of central carbon, amino acid and nucleotide metabolism. With these methods we also include pathway branch points that are important diversions from the central carbon metabolism. The methods presented were optimized using *Saccharomyces cerevisiae* cells, but are widely applicable to any cell type.

In recent studies, we have measured stable-isotope label incorporation into amino acids and nucleotides, under different growth conditions and genetic backgrounds ^10, 11^. Here we summarize the methodology for analyzing steady-state amounts of these and central carbon metabolism pathway metabolites by LC-MS/MS, using multiple-reaction monitoring (MRM) methods. We also use the same method for designing and executing stable isotope-based labeling experiments for MFA. Lastly, we discuss important nuances of labeling experiments for MFA, and point to some of the expected kinetics of label incorporation. These simple, versatile methods can be utilized towards gaining useful insights into synthesis of metabolites in any cell type.

## Methods and results

**Figure 1.**
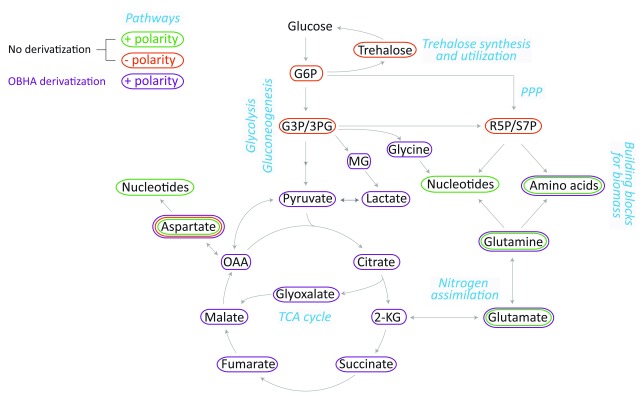
Metabolites and pathways covered in the presented LC-MS/MS methods. Metabolites from the indicated pathways covering central carbon metabolism and their branches could be measured with the use of different gradients, and a derivatization method (discussed in the main text), using a single HPLC column. Standards were used for developing multiple reaction monitoring (MRM) parameters. All abbreviations used and MRM parameters are listed in Table 1.

The metabolites were measured using Synergi 4µ Fusion-RP 80A column (100 /150× 4.6 mm, Phenomenex) by either of the methods described below. The choice of this type of column was specific. Typically many LC methods use C-18 based columns ^1, 4, 8, 9^, which do not resolve polar or charged metabolites unless ion-pair coupling reagents are used. However, ion pair coupling reagents can cause clogging of HPLC lines and pumps, as well as the injectors in the mass spectrometer. The use of the Fusion-RP column allows resolution of several charged metabolites (as described), particularly after simple derivatization, and negates the need of ion-pair coupling reagents. As illustrated in Figure 1, with the described methods, we can cover central carbon metabolism pathways, along some branches that emanate from them. We can also measure all amino acids, and nucleotides, which form the majority of the biomass in a growing cell.

**Table 1.** LC-MS/MS parameters used for quantitation of steady-state levels of metabolites.

Metabolite	Q1/Q3 (Parent/Product)	CE	RT	Comments
**No derivatization (positive polarity)**				
*Amino acids*				
Lysine (K)	147.0 / 84.1	16	2.72	Amino acid pairs where Q1/Q3 are identical (K and Q, I and L) could be separated by HPLC, allowing unambiguous detection. Glycine could not be detected by this method.
Histidine (H)	156.0 / 110.0	12	2.76	
Arginine (R)	175.2 / 60.0	14	2.79	
Serine (S)	106.0/60.0	13	3.27	
Alanine (A)	90.0/44.0	11	3.30	
Asparagine (N)	133.1 / 74.0	15	3.30	
Glutamine (Q)	147.0 / 84.1	16	3.36	
Threonine (T)	120.0/56.0	30	3.39	
Aspartate (D)	134.1/74.0	15	3.46	
Glutamate (E)	148.1/84.1	16	3.48	
Cysteine (C)	122.2/59.0	27	3.58	
Proline (P)	116.0 / 70.0	11	3.84	
Valine (V)	118.0/72.0	15	4.35	
Methionine (M)	150.1/56.0	15	5.30	
Isoleucine (I)	132.0/86.0	11	6.53	
Leucine (L)	132.0/86.0	11	6.90	
Tyrosine (Y)	182.2 / 136.0	14	7.04	
Phenylalanine (F)	166.0 / 120.0	25	9.12	
Tryptophan (W)	205.0 / 146.0	16	10.14	
*Nucleotides*				
Adenosine 5'- monophosphate (AMP)	348/136	21	6.62	Release of nitrogen base monitored.
Guanosine 5'- monophosphate (GMP)	364/152	18	7.96	
Cytidine 5'-monophosphate (CMP)	324/112	21	4.57	
Uridine 5'-monophosphate (UMP)	325/113	21	7.83	
*Miscellaneous (methionine and cysteine metabolism related)*				
S-Adenosyl methionine (SAM)	399/250	13	2.86	
S-Adenosyl homocysteine (SAH)	385/136	19	6.92	
Glutathione reduced (GSH)	308/162	17	6.05	
Glutathione oxidized (GSSG)	613/231	34	7.45	
**No derivatization (negative polarity)**				
Aspartate (D)	131.9/88.1	-20	3.26	To monitor fate of aspartate into gluconeogenesis.
Trehalose (Tre)	341.3/179.3	-17	4.00	
*Sugar phosphates (Glycolysis and PPP)*				
Glucose 6-phosphate (G6P)	259/97	-20	3.14	Caution: The RTs are very close to each other for sugar phosphates here. One can use shallow gradients or specialized methods to resolve these compounds (refer text).
Glyceraldehyde 3- phosphate (G3P)	169/97	-20	3.17	
3-Phosphoglycerate (3PG)	185/97	-20	3.02	
Ribose 5-phosphate (R5P)	229/97	-20	3.20	
Sedoheptulose 7- phosphate (S7P)	289/97	-20	3.15	
**OBHA derivatization (positive polarity; ^1^Gradient 1, ^2^Gradient 2 (see text))**				
*Glycolysis-derived intermediates*				
Pyruvate (Pyr)	299/181	15	9.43 ^1^	
Lactate (Lac)	196/91.2, 196/124, 196/65.2	25, 15 40	6.71 ^1^	
Methylglyoxal (MG)	283/91.2, 283/158.2	25, 14	11.30 ^1^	
*Amino acids*				
Glycine (G)	181/166	17	8.9 ^2^	
Aspartate (D)	344/91.2	32	5.58 ^2^	
Glutamate (E)	253/91.2	21	11.9 ^2^	
Glutamine (Q)	251/91.2	34	8.04 ^2^	
*TCA cycle intermediates*				
Citrate (Cit)	508/91.2, 508/385	25, 7	8.70 ^1^	
2-Ketoglutarate (2-KG)	462/91.2, 462/339	25, 11	9.34 ^1^	
Succinate (Suc)	329/206, 329/91.2	15, 34	8.60 ^2^	
Fumarate (Fum)	327/204, 327/91.2	15, 34	9.25 ^2^	
Malate (Mal)	345/91.2	33	8.07 ^2^	
Oxaloacetate (OAA)	448/325	10	9.20 ^1^	
Glyoxalate	285/65.1, 285/91.2	51, 33	9.37 ^2^	
2-Hydroxyglutarate (2-HG)	359/91.2	35	7.8 ^2^	

The protocols for metabolite extraction and derivatization (whenever required), along with LC-MS/MS parameters are given below. Essentially, changes in the LC gradient and polarity of the instrument were applied to measure amino acids, nucleotides, trehalose and sugar phosphates. A pre-column derivatization method was required for reliable detections and estimations of the TCA cycle metabolites, methylglyoxal, pyruvate and glyoxalate. The same derivatization method can also be applied to amino acids as well.

### Metabolite extraction

The metabolites were extracted as described elsewhere ^12^ with slight modifications. The protocol presented was followed for metabolite extraction from *Saccharomyces cerevisiae*. However, this method (excluding step 2 and 3, and using step 4) works for typical mammalian cell lines, such as HEK293 or isolated mouse T-lymphocytes.

1) Yeast cells were grown in the experimental condition and harvested during the log phase (OD _600nm_ of 0.8-1.4).

2) Cells were quenched for 5 min in 4 volumes of 60% methanol (maintained at -45°C) and then centrifuged at 1000g (-5°C).

3) The pellet was re-suspended in 700 µl of 60% methanol (maintained at -45°C) and then centrifuged at 1000g (-5°C).

4) The pellet obtained was re-suspended in 1 ml 75% ethanol and kept at 80°C for 3 min, immediately followed by incubation on ice for 5 min and centrifugation at 20000g for 10 min.

5) The final supernatant was dried on a vacuum concentrator for 3–4h and then stored at -80°C till further use.

At the time of metabolite measurement, the extract was dissolved in a suitable solvent (see below) and injected into the Synergi Fusion-RP column on Agilent’s 1290 infinity series UHPLC system coupled to a triple-quadrupole type mass spectrometer (Sciex QTRAP 6500, or Thermo TSQ Vantage). Standards were used for developing multiple reaction monitoring (MRM) methods.

### Steady-state metabolite measurements without derivatization

**a) Positive polarity mode:**

*Methodology and chromatograms*

For reliable detection of amino acids and nucleotides in positive polarity mode, metabolite extract was dissolved in 20–50% methanol with 0.1% formic acid. The buffers and the gradient used for separation: buffer A (aqueous phase), 99.9% H _2_O/0.1% formic acid; and buffer B (organic phase), 99.9% methanol/0.1% formic acid (column temperature, 40°C; flow rate, 0.4 ml/min; T = 0 min, 0% B; T = 3 min, 5% B; T = 10 min, 60% B; T = 11 min, 95% B; T = 14 min, 95% B; T = 15 min, 5% B; T = 16 min, 0% B; T = 21 min, stop). The area under each peak was calculated using either AB SCIEX MultiQuant software 3.0.1 or Thermo Xcalibur software 2.2 SP1.48 (Qual and Quan browsers).

**Figure 2.**
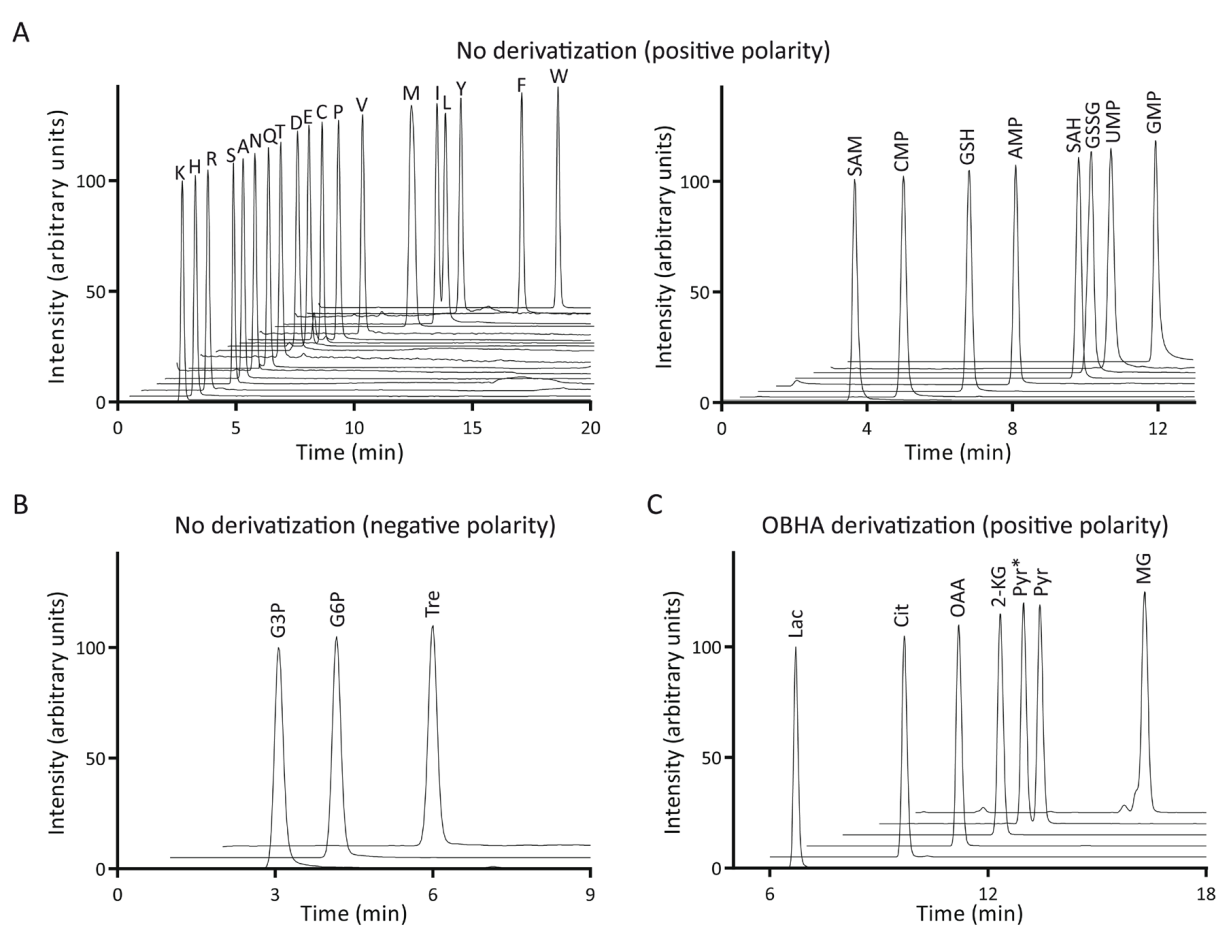
Chromatograms of select metabolites detected using different methods. ( **A**) The left panel shows chromatograms of all amino acids, except glycine. The right panel covers nucleotides and a few methionine/cysteine related metabolites. ( **B**) Sugar phosphates and trehalose were detected in negative polarity. A select set of chromatograms is shown here. However, this method does not give good separation of sugar phosphates from one another, and can be improved using a shallow gradient. ( **C**) Metabolites with carbonyl/carboxyl functional groups from glycolysis and the TCA cycle, as well as some of their extension pathways, were derivatized with OBHA, and select sets of LC-MS/MS chromatograms are shown. In the standard pyruvate sample, two peaks (indicated as Pyr and Pyr*) were observed; the identity of these peaks remains unclear. Note that all intensity values (except for leucine) were normalized to 100, for the ease of visualization. All abbreviations used here are listed in Table 1.

Amino acids (except glycine) and nucleotides could be easily separated with the given gradient ( Figure 2A, left panel; Table 1). Separation of amino acid pairs where parent mass (Q1) and the fragment masses (Q3) are identical (isoleucine and leucine, lysine and glutamine) could also be resolved on the column. With the given parameters, we could also detect cysteine and methionine related metabolites such as S-adenosyl methionine (SAM), S-adenosyl homocysteine (SAH), glutathione reduced (GSH) and glutathione oxidized (GSSG) ( Figure 2A, right panel; Table 1).

**b) Negative polarity mode:**

*Methodology and chromatograms*

For detection of trehalose, triose phosphates and sugar phosphates, the metabolite extract was dissolved in 20–50% acetonitrile with 5 mM ammonium acetate. For negative polarity mode, buffers used for separation: buffer A, 5 mM ammonium acetate in H _2_O; and buffer B, 100% acetonitrile (column temperature, 25°C; flow rate: 0.4 ml/min; T = 0 min, 0% B; T = 3 min, 5% B; T = 10 min, 60% B; T = 11 min, 95% B; T = 14 min, 95% B; T = 15 min, 5% B; T = 16 min, 0% B; T = 21 min, stop).

We could resolve trehalose from other sugar phosphates ( Figure 2B). However, resolving individual sugar phosphates on this column is challenging and can be attained by narrowing the elution gradient. It should be noted that a different column ^13^, or a derivatization-based method ^3^ should be considered if resolving the sugar phosphates is the primary goal of the study.

### Steady-state metabolite measurements with derivatization

*Methodology for OBHA derivatization and chromatograms*

Metabolites from glycolysis and the TCA cycle that have functional carbonyl and carboxyl groups have been shown to be robustly derivatized with *O*-benzylhydroxylamine (OBHA) ^2^. We optimized this method of derivatization and LC-MS/MS-detection with several modifications. This following protocol was used for derivatization:

1) The metabolite extract was dissolved in 20–100 µl of water and 50 µl of 1M 1-ethyl-3-(3-dimethylaminopropyl) carbodiimide (EDC; prepared in pyridine buffer, pH 5.0), and was mixed by shaking (avoid hard vortexing).

2) 100 µl of 0.5M OBHA (prepared in pyridine buffer, pH 5.0) was added to the above mixture and mixed by shaking for 1 h.

3) 300 µl of ethyl acetate was added to the reaction mixture and mixed by shaking for 10 min.

4) The top layer was extracted in a new vial. Ethyl acetate extraction was done two more times and the top layers were mixed together.

5) The derivatized extract was dried using a vacuum concentrator for 3–4h and then stored at -80°C till further use. The vacuum-dried sample was dissolved in 1 ml of 1:1 water: methanol and a suitable dilution (typically in the range of 1:10 to 1:100) was injected into the Synergy column (150 mm) and further derivatized products were detected on the mass spectrometer (see Table 1 for Q1/Q3 parameters).

The buffers and gradients used for separation: buffer A, 99.9% H _2_O/0.1% formic acid; and buffer B, 99.9% methanol/0.1% formic acid (column temperature, 40°C; flow rate, 0.4 ml/min; Gradient 1: T = 0 min, 50% B; T = 2 min, 75% B; T = 6 min, 100% B; T = 15 min, 100% B; T = 17 min, 50% B; T = 21 min, stop; Gradient 2: T = 0 min, 50% B; T = 2 min, 65% B; T = 12 min, 90% B; T = 12.01 min, 100% B; T = 15 min, 100% B; T = 20 min, 50% B; T = 26 min, stop).

With the given gradients, we could detect several additional metabolites from glycolysis and the TCA cycle than reported earlier ^2^. We also cover important branch-point metabolites such as methylglyoxal and glyoxalate ( Figure 2C; Table 1). Finally, we could also detect amino acids with OBHA derivatization, and observed that the sensitivity of detection of amino acids is at least 2 orders of magnitude greater than the non-derivatized protocols.

### Analysis of *de novo* biosynthesis of metabolites by using stable isotope-enriched metabolic precursors

*a) Experimental set-up*

**Figure 3.**
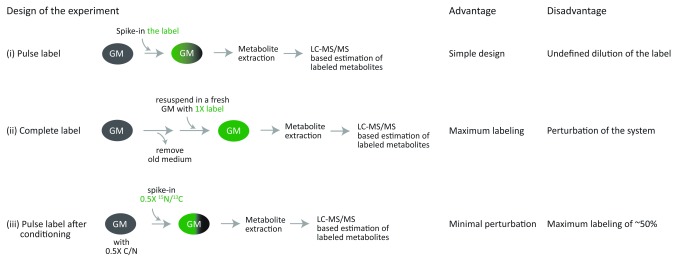
Different approaches for stable isotope based labeling experiments. Three designs of stable-isotope labeling experiments are shown, along with their advantages and dis-advantages: (i) Pulse labeling, where the cells are suddenly exposed to the labeled precursor and thus its incorporation depends upon the amounts of unlabeled precursor present in their current growth medium, (ii) Removing the growth medium and re-suspending the cells with the fresh medium that has labeled metabolite ensured maximal labeling, and (iii) Conditioning the cells with a growth medium with 0.5X metabolite precursor and then pulse labeling with the remaining 0.5X as the labeled precursor. This approach allows approximately 50% or more labeling at the steady-state, depending on the rate of usage of the metabolite and duration of conditioning.

As changes in the steady-state levels of metabolites is the net result of its synthesis and utilization, for understanding metabolic flux or new synthesis it is important to utilize stable isotope-enriched metabolic precursors such as ^13^C-glucose or ^15^N-ammonim sulfate, and observe the label ( ^13^C or ^15^N) incorporation into the metabolites of interest. Three strategies can be adopted for the labeling experiments, which offer specific advantages and disadvantages (as illustrated in Figure 3): (i) the simplest approach is to spike-in the labeled precursor exogenously in the growth medium. However, this may result in dilution of the label if the same precursor is also available in the growth medium. (ii) one can remove the old growth medium and add fresh medium that has labeled precursor, with the caveat that this might perturb the system under study. (iii) the third approach that we have found especially useful is to allow cells to grow in a medium with half the concentration required of that particular precursor, and spike-in the remaining half as the labeled fraction after a few hours of growth. This approach allows minimal perturbation of the system, with a maximum possible label incorporation of ~50% (initial) for the downstream metabolite. Two experiments, which use the third experimental design, involving ^13^C- and ^15^N-metabolic precursors and their utilization for generation of a nucleotide (a complex metabolite) are discussed in the next section.

*b) Methods of analysis*

**Figure 4.**
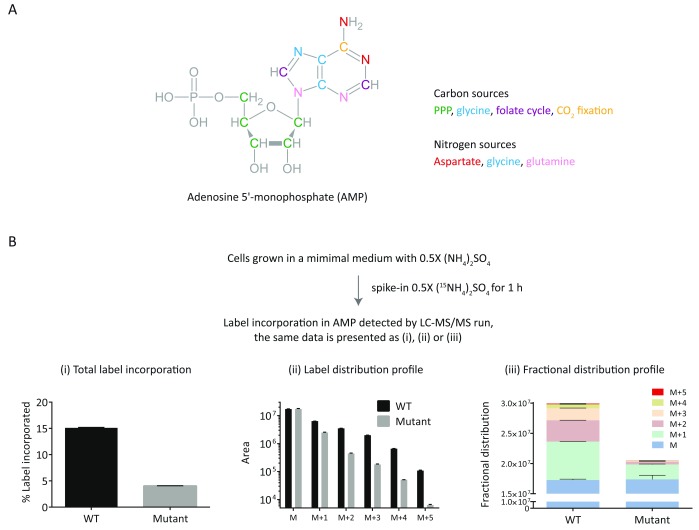
Different ways of presenting and analyzing stable-isotope label incorporation data for complex metabolites. ( **A**) The structure of AMP is shown along with the pathway-source of individual carbon and nitrogen atoms. The labeling of this metabolite would involve multiple inputs from the respective pathways. ( **B**) The label incorporation from ^15^N-ammonium sulfate into AMP was followed as indicated. The obtained data is presented in different formats (i, ii and iii). “M” represents the unlabeled metabolite and M+(1 to 5) indicate label accumulation of 1 to 5 ^15^N atoms in the metabolite. The MS parameters used for label detection are given in Table 2.

We monitored ^15^N incorporation in the nitrogen base of adenosine 5'-monophosphate (AMP). AMP is generated by incorporating inputs from multiple pathways ( Figure 4A). As the AMP detection monitors loss of the nitrogen base ( Table 1), it was straightforward to predict the Q1/Q3 parameters for the labeled moieties of AMP ( Table 2). It should be emphasized here that the comparison of label incorporation (in different media conditions or genetic backgrounds) should be done before a steady-state level of label is achieved and a pilot experiment suggestive of the kinetics of labeling is often helpful in designing the final experiments.

**Table 2.** LC-MS/MS parameters used for ^13^C and ^15^N labeling experiments.

Metabolite	Q1/Q3 (Parent/ Product)	Comments
**^15^N labeling experiment**		
AMP	348/136	Q3 has all nitrogen atoms.
^15^N_AMP_1	349/137	
^15^N_AMP_2	350/138	
^15^N_AMP_3	351/139	
^15^N_AMP_4	352/140	
^15^N_AMP_5	353/141	
**^13^C labeling experiment**		
AMP	348/136	Q3 has 5 carbons coming from the nitrogen base. The rest of the carbons form the ribose ring.
^13^C_AMP_1	349/137	
^13^C_AMP_2	350/138	
^13^C_AMP_3	351/139	
^13^C_AMP_4	352/140	
^13^C_AMP_5	353/141	
^13^C_AMP_5_with labeled ribose	353/136	
^13^C_AMP_6	354/137	
^13^C_AMP_7	355/138	
^13^C_AMP_8	356/139	
^13^C_AMP_9	357/140	
^13^C_AMP_10	358/141	

The labeling data can be presented in different ways, where different aspects of labeling are highlighted. As shown in Figure 4B, the data could be presented as (i) percent label incorporation which gives an idea about overall label incorporation, (ii) plotting individual peaks from M+1 to M+5 informs about the labeling pattern or blocks in label incorporation, and (iii) a fractional distribution profile can be used for comparing the total amounts as well as fractional contribution of individual labels.

*c) Kinetics of labeling*

**Figure 5.**
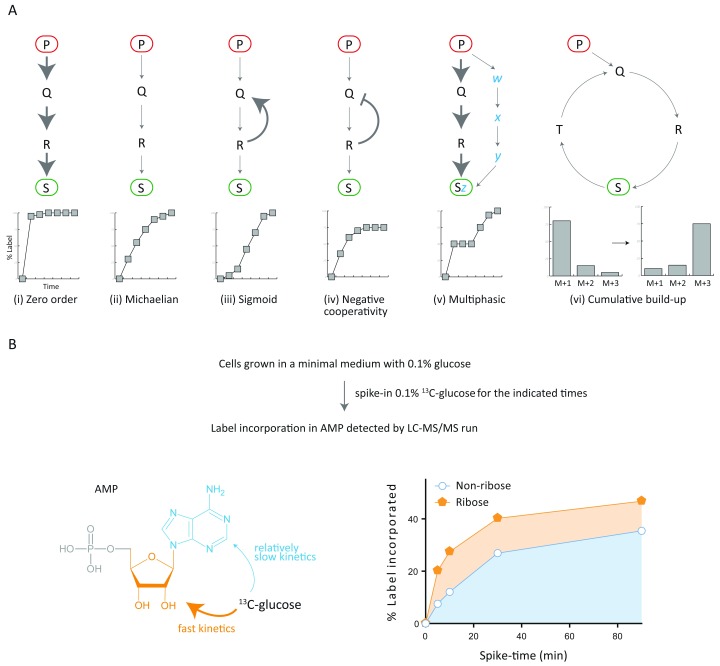
Kinetics of label incorporation in complex metabolites. ( **A**) Different examples of the kinetics of label incorporation are presented, considering the characteristics of the pathway by which the metabolite of interest is synthesized. The expected influence of the flux (cases i and ii), regulation (cases iii and iv), number of inputs (case v), or structure (cases i-v versus case vi) of the pathway onto the kinetics of label incorporation is presented. The stable-isotope label is fed in the form of metabolite precursor P (red oval) and the label is detected in S (green oval). The corresponding graphs are not data points and represent predicted kinetic patterns. ( **B**) ^13^C label incorporation in AMP from ^13^C-glucose was followed for the indicated time points. The data indicates that the ribose moiety in AMP is incorporated much faster than the non-ribose part. The MS parameters used for label detection are given in Table 2.

As mentioned earlier, in many cases it is useful to compare the label incorporation before a steady-state of labeling is reached. However, monitoring or comparing the kinetics of label incorporation itself can give additional information about the biosynthetic pathway(s) involved. Depending on the metabolite of our interest, the biological pathway that generates the metabolite could have differences in its organization (linear or cyclic), flux (high or low) and/or regulation (positive or negative cooperativity). The metabolite could also be a result of one or more pathways that have distinct features (as mentioned above). These different possibilities are covered in Figure 5A.

To illustrate how monitoring the labeling kinetics in-depth is useful, we present the ^13^C label incorporation into AMP as an example. The carbon skeleton of AMP can be divided into two parts i.e. ribose moiety and the nitrogen base (non-ribose part). We successfully monitored the kinetics of label incorporation in two parts of the molecule. We could estimate the label incorporation into these two distinct parts using the distinct Q1/Q3 parameters mentioned in Table 2. The analysis clearly showed that the ribose part of the molecule is labeled rapidly, as compared to the nitrogen base ( Figure 5B) when the label is obtained from ^13^C-glucose. One can further tease out the individual contribution of all the pathways ( Figure 4A) that result in the biosynthesis of AMP, by repeating the experiment with a different start-point label and analyzing the kinetics and distribution of M+ intensities.

## Discussion

Combining information from other “omics” (such as transcriptomics, genomics) with metabolomics can be a very powerful approach in determining how different aspects (such as signaling pathways, genes) are connected to metabolism ^10, 11, 14, 15^. Although various LC-MS/MS based methods are available for quantitation of metabolites, they rarely cover multiple pathways. Here we show that by changing the LC parameters, and by using a derivatization based method, multiple branch-points from the central carbon metabolism could be covered ( Figure 1). In developing these methods, we specifically avoided the use of ion-pairing agents because of the problems associated with their usage. For developing this comprehensive set of methods, we purposefully chose a column that has affinity for both polar and non-polar molecules (the stationary phase being polar embedded C18 with trimethylsilyl end capping). Any other similarly designed column may also be used in such analysis. We observed that without any derivatization, amino acid and nucleotide biosynthesis as well as the flux in gluconeogenesis and trehalose metabolism could be followed ( Figure 1). The OBHA derivatization was found to be a robust, highly sensitive method for detecting TCA metabolites, amino acids and a few glycolytic intermediates ( Figure 2).

Multiple good resources are available for metabolic flux analysis (MFA) with ^13^C-glucose as the input ^16, 17^. Although many studies have looked at central carbon metabolism using labeled glucose, different molecules can be biosynthesized from these pathways via a variety of transformations. However, as demonstrated in Figure 4 and Figure 5, complex metabolites like adenosine 5'-monophosphate (AMP) have inputs from multiple pathways, and MFA in these metabolites is tricky or beyond the scope of most of the laboratories. For such cases, one can design simple but informative experiments ( Figure 3– Figure 5), where we monitor incorporation of the label into the complex molecule of interest.

The analysis of ^13^C-glucose incorporation in AMP ( Figure 5) points towards the need to de-convolute the kinetics patterns from labeling experiments. An in-depth analysis will facilitate useful insights into the system under study, such as identifying the precursor for faster label incorporation, contribution of different pathways to make a given metabolite, *et cetera*. This will allow us to understand the dynamics of multiple pathways and the hierarchy therein.

## Data availability

The raw data is deposited at OSF under the file name “Mass spec data for WOR method article_2018”: https://doi.org/10.17605/OSF.IO/KV6FA ^18^.

Data are available under the terms of the Creative Commons Zero "No rights reserved" data waiver (CC0 1.0 Public domain dedication).
