# Supplementary material for: A versatile LC-MS/MS approach for comprehensive, quantitative analysis of central metabolic pathways
Source: Wellcome Open Res. 2018 Sep 20;3:122. [Version 1] doi: 10.12688/wellcomeopenres.14832.1 (PMC6171562; doi:10.12688/wellcomeopenres.14832.1)
Supplement: Peer review history

# Peer review: Referee response for version 1

**DOI:** 10.21956/wellcomeopenres.16161.r33962

**Published:** 2018-09-28

**Authors:** Vinay Bulusu, Giovanny Rodriguez Blanco

**Affiliations:** 1 Beatson Institute for Cancer Research (Cancer Research UK Beatson Institute), Glasgow, UK

The manuscript by Adhish Walvekar, Sunil Laxman and colleagues describe single column LC MS/MS methods for steady state metabolomics and stable isotope tracing. The rationale for developing these methods using Fusion-RP column is to prevent using ion pairing agents and to allow better separation of metabolites. The methodology is described primarily for yeast cells and the authors claim that the method can be used for mammalian cells as well. Overall the methodology is very well described and will surely add nicely to the existing methods used in metabolomics. However, the authors should consider the following points to further strengthen the manuscript.

 1. Are the peak intensities of the metabolites in the linear dynamic range? This can be tested by injecting different dilutions of the extract and checking the linearity of the signal intensity. The authors should mention the amount of starting biomass and/or optimal working dilution.

 2. In the extraction method, have the authors taken care of the stability of metabolites? This should be addressed especially as extraction is done in hot ethanol and there is a 3-4h vacuum drying step. The authors should indicate which metabolites are potentially unstable during extraction and caution should be exerted in their analysis.

 3.  Most of the data except figure 4B and figure 5B is qualitative. The authors should consider showing calibration curves for at least one metabolite from each class (amino acid, nucleotide, sugars). 

 4. For steady state metabolomics, we routinely use internal standards (such as 13C amino acids) in each run to account for matrix effects. The authors should mention how they account for matrix effects especially when they want to compare multiple samples.

 5. The authors use triple quadrupole mass spectrometers in MRM mode, which is great in terms of sensitivity, but are limited due to their targeted nature. This makes them less attractive to be used in stable isotope tracing experiments. As shown in table 2, the instruments have to be programmed for specific parent/product (Q1/Q3) scan events. This very much limits the coverage of metabolites in a single run in comparison to using high-resolution ion trap mass spectrometers in full scan mode. The authors should probably mention this limitation in their manuscript. 

 6. It will be good for people outside metabolomics field, if authors mention time of labelling in reference to figure 3. Different pathways have different time frames to achieve metabolic steady state. Glycolysis in minutes, TCA cycle in hours, nucleotide synthesis and fatty acid synthesis in days. This could be mentioned in the text.

 7. Figure 4B (ii): If this is label distribution, then each isotopologue peak area should be expressed as a fraction of the total peak area. 

 (iii): is the y-axis normalized to biomass (cell number/protein)?

We have read this submission. We believe that we have an appropriate level of expertise to confirm that it is of an acceptable scientific standard.

# Peer review: Referee response for version 1

**DOI:** 10.21956/wellcomeopenres.16161.r33965

**Published:** 2018-09-24

**Authors:** Siddhesh S. Kamat

**Affiliations:** 1 Department of Biology, Indian Institute of Science Education and Research (IISER), Pune, Maharashtra, India

There is an increasing need for quantitative LC-MS/MS methods that are global and faster, and use a single method to detect and quantify, different metabolites from several classes (e.g. sugars, amino acids etc.). In this manuscript, the authors describe how using a single LC-method and column separation, several metabolites that include important TCA intermediates, sugars, amino acids, and important biological cofactors (SAM, SAH, alpha-KG) can be assessed in tandem in a single run (more or less). Additionally the authors describe the use of OBHA in making amino acid derivatives that are extremely useful in their quantitative estimations using LC-MS/MS. The authors also describe different stable isotope labelling methods, and its implication in metabolite assessment using the LC-MS/MS method described in this manuscript. The article is very well written and the results are interpreted clearly in the context of the LC-MS/MS method described here. In all, the manuscript is of very good quality, and will definitely benefit the metabolomics community at large. There are however, a few things that the authors might consider that could add further value to this manuscript:

 1. Some MS parameters are missing for the LC-MS/MS methods described (e.g. declustering potential, entrance potential etc.). It would be good to report this in the manuscript, especially since the authors are using two MS instruments, and these parameters could be added to the manuscript for better reproduction of this data. It would also be good to clarify whether the CE values reported in the manuscript is the same for both instruments or there are subtle differences in this value for different instruments? 

 2. It might also be good to report the LOD-LOQ for different metabolites for this particular method to understand the sensitivity and linear dynamic range for the different metabolites reported for this method. 

 3. It might be nice as part of this manuscript, to report an extraction procedure and downstream LC-MS/MS analysis for the metabolites discussed for one or perhaps two mammalian cell lines (e.g. HEK293T and/or HeLa and/or human T-cells), as this will certainly benefit the metabolomics community beyond those working with yeast cells. 

 4. Analogous to iTRAQ and TMT, perhaps the authors can think of adapting the OBHA derivative strategy to heavy and light labelling to enable direct quantitation of amino acids between two samples (e.g. control versus treated).... that way fewer replicates might be needed for doing such a comparative analysis, and the need for internal standards and instrument calibration might be circumvented. I am not sure about the feasibility of this though? Perhaps the authors can elaborate on the feasibility of this?

 5. For the current Fig. 1 and 3: it might be good to change the colour scheme to make contrasting colours, so that different metabolites and schemes stand out better in these figures. 

 6. For Figure 4A: make atoms font bold so that colours stand out better

I have read this submission. I believe that I have an appropriate level of expertise to confirm that it is of an acceptable scientific standard.
